# P-Type ATPase Apt1 of the Fungal Pathogen *Cryptococcus neoformans* Is a Lipid Flippase of Broad Substrate Specificity

**DOI:** 10.3390/jof7100843

**Published:** 2021-10-08

**Authors:** Lyubomir Dimitrov Stanchev, Juliana Rizzo, Rebecca Peschel, Lilli A. Pazurek, Lasse Bredegaard, Sarina Veit, Sabine Laerbusch, Marcio L. Rodrigues, Rosa L. López-Marqués, Thomas Günther Pomorski

**Affiliations:** 1 Department of Molecular Biochemistry, Faculty of Chemistry and Biochemistry, Ruhr University Bochum, 44780 Bochum, Germany; lydist@biosustain.dtu.dk (L.D.S.); Rebecca.Peschel@ruhr-uni-bochum.de (R.P.); lilli.pazurek@ruhr-uni-bochum.de (L.A.P.); sarina.veit@ruhr-uni-bochum.de (S.V.); sabine.laerbusch@ruhr-uni-bochum.de (S.L.); 2 Department of Plant and Environmental Sciences, University of Copenhagen, Thorvaldsensvej 40, 1871 Frederiksberg, Denmark; gjh338@alumni.ku.dk (L.B.); rlo@plen.ku.dk (R.L.L.-M.); 3 Instituto de Microbiologia Paulo de Góes (IMPG), Universidade Federal do Rio de Janeiro (UFRJ), Rio de Janeiro 21941-902, Brazil; juliana.rizzob@gmail.com (J.R.); marcio.rodrigues@fiocruz.br (M.L.R.); 4 Unité Biologie des ARN des Pathogènes Fongiques, Département de Mycologie, Institut Pasteur, 75015 Paris, France; 5 Instituto Carlos Chagas, Fiocruz, Curitiba 81310-020, Brazil

**Keywords:** β-subunit, CDC50 protein, heterologous expression, lipid transport, membrane transport protein, P4-ATPase

## Abstract

Lipid flippases of the P4-ATPase family are ATP-driven transporters that translocate lipids from the exoplasmic to the cytosolic leaflet of biological membranes. In the encapsulated fungal pathogen *Cryptococcus neoformans*, the P4-ATPase Apt1p is an important regulator of polysaccharide secretion and pathogenesis, but its biochemical characterization is lacking. Phylogenetic analysis revealed that Apt1p belongs to the subclade of P4A-ATPases characterized by the common requirement for a β-subunit. Using heterologous expression in *S. cerevisiae*, we demonstrate that Apt1p forms a heterodimeric complex with the *C. neoformans* Cdc50 protein. This association is required for both localization and activity of the transporter complex. Lipid flippase activity of the heterodimeric complex was assessed by complementation tests and uptake assays employing fluorescent lipids and revealed a broad substrate specificity, including several phospholipids, the alkylphospholipid miltefosine, and the glycolipids glucosyl- and galactosylceramide. Our results suggest that transbilayer lipid transport in *C. neoformans* is finely regulated to promote fungal virulence, which reinforces the potential of Apt1p as a target for antifungal drug development.

## 1. Introduction

*Cryptococcus neoformans* is an encapsulated fungal pathogen that causes severe infection of the central nervous system. The pathogenicity of *C. neoformans* is largely dependent on secretory mechanisms, which results in the transport of important virulence factors to the extracellular space, including fungal melanin, extracellular enzymes (e.g., laccase, urease, and phospholipase B1), and immunomodulatory polysaccharides [1]. Cryptococcal extracellular polysaccharides such as glucuronoxylomannan are considered important regulators of pathogenicity [2] and are required for capsule formation, which protects the fungus against host immune mechanisms (reviewed in reference [3]). Secretion of extracellular molecules is a highly regulated process relying on intense membrane remodeling and involving hundreds of different cellular proteins. Among these are members of the P4 subfamily of P-type ATPases (P4-ATPases), which are lipid flippases that catalyze the translocation of lipids from the exoplasmic to the cytosolic leaflet of cell membranes [4,5]. Besides generating and maintaining the asymmetric distribution of lipids in biological membranes, P4-ATPases directly or indirectly affect a host of cellular functions, including vesicle biogenesis, both at the plasma membrane [6] and in the trans-Golgi network [7,8]. Flippases have also been linked to extracellular vesicle formation [9,10,11].

Most P4-ATPases associate with an accessory subunit known as Cdc50/Lem3 protein, resulting in a heterodimeric complex. This association is required for both proper localization and activity of the flippase complex [12,13] but seems not to affect its substrate specificity [14]. Recent cryo-electron microscopy structures of four P4-ATPases in complex with their respective Cdc50 subunits have revealed the structural basis of P4-ATPase-Cdc50 protein association [15,16,17,18,19,20]. Genes encoding P4-ATPases are highly conserved in evolution, and members of this P-type ATPase subfamily have been found in genomes of unicellular eukaryotes, plants, and animals. The nonpathogenic yeast *S. cerevisiae* expresses five P4-ATPases, including *Sc*Neo1p in the endosomal membranes, *Sc*Drs2p and *Sc*Dnf3p mostly in the trans-Golgi network, and *Sc*Dnf1p and *Sc*Dnf2p at the plasma membrane [6,7,21,22].

While initially characterized as aminophospholipid translocases flipping phosphatidylserine (PS) and -ethanolamine (PE) across cellular membranes, recent studies of individual P4-ATPase family members show that P4-ATPases differ in their substrate specificities and can mediate transport of a broader range of lipid substrates. Of the *S. cerevisiae* flippases, *Sc*Drs2p is a rather specific PS transporter [23,24], whereas *Sc*Dnf1p, *Sc*Dnf2p, and *Sc*Dnf3p can transport PS, PE, and phosphatidylcholine (PC) to different extents [6,25,26,27,28]. In addition, *Sc*Dnf1p and *Sc*Dnf2p can also recognize lysophospholipids, alkylphospholipids, sphingomyelin (SM), and glucosyl- and galactosylceramide (GlcCer and GalCer) as substrates [29,30,31,32,33,34]. *Sc*Neo1 is apparently implicated in the transport of PE and PS, but the lipid substrate for this P4-ATPase remains to be confirmed [35]. 

The genome of *C. neoformans* contains four putative P4-ATPases, designated Apt1–4p, and one putative Cdc50 protein. It was recently shown that loss of *APT1* results in disturbed glucuronoxylomannan secretion, increased cryptococcal susceptibility to antifungal agents, reduced fungal survival inside macrophages, and attenuated fungal virulence in a murine infection model [36,37,38]. Similarly, gene disruption of cryptococcal Cdc50 protein affects glucuronoxylomannan secretion, iron acquisition, sensitivity to caspofungin, susceptibility to macrophages, and virulence in a mice model of cryptococcosis [39,40]. However, the *Cryptococcus*-encoded P4-ATPases in complex with the potential Cdc50 subunit remain as putative transporters waiting for functional characterization. In this study, we provide the first functional characterization of the *Cryptococcus* lipid flippase Apt1p, an emerging antifungal target candidate.

## 2. Materials and Methods

Materials. PageRuler^TM^ pre-stained Thermo Scientific (Waltham, Massachusetts, USA) was used for size determination in SDS-PAGE gels. PC (#850457), PS (#840034), and lipids tagged with nitrobenzoxadiazole (NBD) were purchased from Avanti Polar Lipids (Alabaster, AL, USA): 1-NBD-dodecanoyl-2-hydroxy-*sn*-glycero-3-phosphocholine (#810128), Palmitoyl-(NBD-hexanoyl)-phosphatidylcholine (#810130), Palmitoyl-(NBD-hexanoyl)-phosphatidylserine (#810192), Palmitoyl-(NBD-hexanoyl)-phosphatidylethanolamine (#810153), Palmitoyl-(NBD-hexanoyl)-phospatidylglycerol (#810164), NBD-hexanoyl-sphingomyelin (#810218), NBD-hexanoyl-glucosylceramide (#810222), NBD-hexanoyl-galactosylceramide (#810220), and NBD-hexanoyl-lactosylceramide (#810226). DNA-modifying enzymes were obtained from New England Biolabs (NEB, Frankfurt am Main, Germany) and Takara Bio USA (Mountain View, CA, USA). Commercial kits for molecular cloning were delivered by New England Biolabs, Macherey Nagel (Düren, Germany) and Zymo Research (Freiburg, Germany). [γ-^32^P]ATP (3000 Ci/mmol) was obtained from Perkin Elmer Inc. (Boston, MA, USA). Unless indicated otherwise, all other chemicals and reagents were obtained from Sigma-Aldrich (München, Germany). Protease inhibitor cocktail contained aprotinin (5 mg), leupeptin (5 mg), pepstatin (5 mg), antipain (25 mg), and benzamidine (785 mg) in 5 mL dimethylsulfoxide and was used at a 1:1000 dilution.

Strains, media, and growth conditions. NEB 5-alpha competent *Escherichia coli* (Invitrogen) was used for all plasmid amplifications and isolations according to standard protocols. Expression and functional complementation were carried out employing the flippase-deficient *S. cerevisiae* mutant strain ZHY709 (MATα *his3 leu2 ura3 met15 dnf1Δ dnf2Δ drs2::LEU2* [21]), with strain BY4741 (MATa *his3 leu2 ura3 met15*; EUROSCARF) as the wild-type strain. Yeast cells were transformed by the lithium acetate method [41] and grown at 30 °C in standard synthetic dextrose (SD) or galactose (SG) medium lacking uracil. For solid media, 2% (*w*/*v*) agar was added. Expression analysis, microscopy, and lipid translocation assays were performed on transformants cultured in selective SG medium at 25 °C and 150 rpm for 26 h [42]. For growth assays, the transformants were pre-cultured in liquid SD medium for 16 h and then diluted with water to 0.2 optical density at 600 nm (OD_600_). Drops (3 μL) of serial 5-fold dilutions were spotted on SD (control) or SG (Induction) plates and incubated at 25 °C for 3 days or at 20 °C for 10 days. When indicated, gradient plates were used, containing maximum concentrations of 140 µg mL^−1^ miltefosin (hexadecylphosphocholine; Calbiochem, La Jolla, CA, USA), 5 µM duramycin (Sigma-Aldrich, St. Louis, MO, USA), 10 µg mL^−1^ perifosine (Sigma-Aldrich, St. Louis, MO, USA), or 15 µg mL^−1^ edelfosine (Avanti Polar Lipids, Alabaster, AL, USA). Cells on gradient plates were dropped at a single OD_600_ = 0.1. All experiments were repeated independently at least three times.

Plasmid Construction. Primers used are listed in Supplementary Table S1. Standard PCR reactions were performed with Q5 High-Fidelity DNA Polymerase (NEB, Ipswich, MA, USA). The wild-type *APT1* coding sequence was originally amplified from H99 *C. neoformans* cDNA corresponding to GenBank entry CNAG_06469 using primer set #1 and introduced into the BamHI restriction site of the pESC-URA vector (Stratagene, La Jolla, CA, USA) using an InFusion Cloning Kit (Takara Clontech), yielding the plasmid pESC-URA-APT1. The plasmid pESC-URA-APT1-myc was generated with oligonucleotides encoding the myc tag and a GSGSGSG linker (primer set #2) using the pESC-URA-APT1 plasmid as a template and a QuickChange Site-Directed Mutagenesis Kit (New England Biolabs). To generate a vector bearing C-terminal green fluorescent protein (GFP)-tagged APT1 (pESC-URA-APT1-GFP), the PCR-amplified *APT1* sequence (primer set #3, pESC-URA-APT1-myc as the plasmid template) and a PCR-amplified *GFP* fragment (primer set #4, pET-SUMO-LUN-GFP plasmid as the template) containing overlapping sequences were introduced into the BamHI restriction site of pESC-URA vector by InFusion cloning. The *CDC50* sequence was amplified from synthetic cDNA (Accession number CNAG_06465) ordered from Integrated DNA Technologies (IDT, Coralville, IA, USA) using primer set #5, and introduced into the NotI site of the corresponding pESC-URA vectors, yielding pESC-URA-APT1-CDC50, pESC-URA-APT1-myc-CDC50, and pESC-URA-APT1-GFP-CDC50. A similar strategy was used to generate pESC-URA-APT1-myc-CDC50-Flag and pESC-URA-APT1-GFP-CDC50-Flag using primer set #6. The point mutant unable to hydrolyze ATP (*apt*^E303Q^) was generated by QuickChange site-directed PCR (primer set #7) following the manufacturer’s instructions (NEB, Ipswich, MA, USA). All constructs were verified by sequencing.

Isolation of total membranes, affinity purification, and immunodetection. Cells harvested by centrifugation (850× *g*, 5 min, 4 °C) were washed twice with ice-cold phosphate-buffered saline (PBS; 130 mM NaCl, 2.6 mM KCl, 7 mM Na_2_HPO_4_, 1.2 mM KH_2_PO_4_, pH 7.4). For Western blot analysis, cells were homogenized using glass beads (0.5 mm, acid washed) in ice-cold lysis buffer I (0.8 M sorbitol, 10 mM EDTA, 50 mM HEPES-KOH, pH 7.2) supplemented with 1 mM phenylmethanesulphonyl fluoride and a protease inhibitor cocktail. After a low-speed centrifugation (500× *g*, 5 min, 4 °C), the supernatant was centrifuged at 100,000× *g* (45 min, 4 °C) to pellet the membranes. The membranes were resuspended in ice-cold lysis buffer I. For purification, cells were lysed in ice-cold lysis buffer II (100 mM NaCl, 20 mM HEPES-NaOH, pH 7.4) supplemented with 1 mM phenylmethanesulphonyl fluoride and the protease inhibitor cocktail. Membranes were collected at 100,000× *g* (45 min, 4 °C), resuspended in glycerol buffer (20% (*w*/*v*) glycerol, 100 mM NaCl, 20 mM HEPES-NaOH, pH 7.4), and homogenized using a Douncer. FLAG-affinity purification was performed essentially as described [43]. Briefly, the membranes were detergent-solubilized at a protein concentration of 1 mg mL^−1^ in glycerol buffer supplemented with 0.6% (*w*/*v*) dodecylmaltoside (DDM) on an end-over-end rotator for 1 h at 4 °C. Insoluble material was removed by centrifugation (100,000× *g*, 60 min, 4 °C), and the supernatant was incubated with 14 µL mL^−1^ anti-FLAG-M2 affinity gel (prewashed twice in glycerol buffer) for 16 h at 4 °C with end-over-end rotation. The gel was washed three times with glycerol buffer containing 0.05% (*w*/*v*) DDM. Proteins were eluted using glycerol buffer containing 0.05% (*w*/*v*) DDM and 400 µg mL^−1^ FLAG peptide. Protein content was determined by DC Protein assay (Bio-Rad Laboratories GmbH, München, Germany). Protein samples were separated on 10% SDS-PAGE gels, transferred to nitrocellulose membranes (Merck Millipore, Milford, MA, USA), and immunodetected with mouse monoclonal antibodies against FLAG (ANTI-FLAG^®^ M2-Antibody, 1:2000; Sigma-Aldrich, St. Louis, MO, USA). Protein blots were probed with alkaline phosphatase-conjugated secondary antibodies (Dako A/S, Glostrup, Denmark) using nitroblue tetrazolium (NBT) and 5-bromocresyl-3-indolyl-phosphate (BCIP) as substrates. In-gel GFP fluorescence was detected with a ChemiDoc™ MP device (Bio-Rad Laboratories GmbH) using the Image Lab™ software and illumination at 488 nm. The purified proteins were quantified by Coomassie R-250 staining on SDS-polyacrylamide gels using bovine serum albumin as a standard. Densitometry analysis of SDS-polyacrylamide gels was performed with a ChemiDoc™ MP device and Image Lab^TM^ Software (Bio-Rad).

Deglycosylation of Cdc50p. Deglycosylation experiments using peptide-N-glycosidase F (PNGAse F, P0704S, New England Biolabs) were carried out according to the manufacturer’s instructions. Briefly, 9 µL purified protein (0.06 µg) as incubated with 1 µL glycoprotein denaturing buffer (10×) for 10 min at 100 °C. After 5 min of incubation on ice, 2 µL glycol-buffer 2 (10×), 2 µL 10% NP-40, and 5 µL ddH_2_O were added. One sample was supplemented with 1 µL PNGase F, and the control sample with 1 µL ddH_2_O and incubated for 1 h at 37 °C. Samples were analyzed by 10% SDS-PAGE followed by Western Blot, as described before.

ATPase Assay. The purified protein was analyzed for ATPase activity using [γ-^32^P]ATP as described by [44]. Briefly, eluates (39 µL, 0.25 µg protein) containing 0.05% (*w/v*) DDM, in the absence or presence of exogenous lipids (0.5 mg mL^−1^) and inhibitor (1 mM orthovanadate) were incubated for 20 min at room temperature for protein activation. Upon addition of 5 µL of an ATP mixture (10 mM ATP, 50 mM MgCl_2_, 20 µCi [γ-^32^P]ATP), ATPase activity was assayed for 60 min at 28 °C. The reaction was stopped by placing the samples on ice and the addition of 1.5 mL of reagent A (10 mM ammonium heptamolybdate in 1 M HCl). The samples were transferred to glass tubes containing 15 µL H_3_PO_4_ (20 mM), and 3 mL of reagent B (isobutanol, cyclohexane, acetone, and reagent A; ratio of 5:5:1:0.1 (*v*/*v*)) was added. After vortexing for 30 s, the samples were incubated for 10 min on ice to allow complete phase separation. Out of the upper phase, 500 µL was taken and added to 2 mL of scintillation fluid (Rotiszint eco plus for hydrophilic samples, Carl Roth, Karlsruhe, Germany) in a scintillation vial, and β-emission was counted for 1 min per sample (2450 MicroBeta2TM, Perkin Elmer, Groningen, The Netherlands). As a reference, 0.2 µL of the ATPase reaction mix in 2 mL scintillation fluid was measured.

NBD-lipid uptake assays, microscopy, and lipid analysis. Uptake experiments were performed at 30 °C for 30 min, essentially as described previously [43]. Flow cytometry was performed on a Becton Dickinson Flow Cytometer equipped with an argon laser using Cell Quest software. Data were analyzed using Cyflogic (CyFlo, Ltd., Turku, Finland), according to [45]. One microgram of propidium iodide in water was added to cells in 1 mL of PBS just before flow cytometry analysis. Data for at least 20,000 cells were collected prior to gating. NBD fluorescence of living cells was plotted on a histogram, and the geometric mean fluorescence intensity was used for further statistical analysis. Fluorescence microscopy and image acquisition were carried out on living cells immobilized on Concanavalin A-coated glass slides using a spectral confocal laser scanning microscope (Leica TCS SP5, Heidelberg, Germany). All images were acquired using a 63×/1.4 numerical-aperture (NA) oil objective. GFP was excited with a 488 nm argon laser, and emission signals were recorded between 500 and 550 nm, DAPI was excited at 355 nm, and emission signals werecollected between 410 and 470 nm. All fluorescent signals were quantified using ImageJ version 1.53c (National Institutes of Health, Madison, WI, USA; downloaded from https://fiji.sc/ (accessed on 15 July 2021)).

Data analysis. Data were analyzed using Excel (Microsoft) and OriginPro (Origin- Lab, Northampton, MA, USA). Data represent means ± s.d. of at least three experiments. 

Phylogenetic analysis. Annotated P4-ATPase protein sequences were retrieved from the Uniprot Database (www.uniprot.org (accessed on 15 July 2021)), except for those corresponding to *Tremella mesenterica*, which were retrieved from NCBI (www.ncbi.nlm.nih.gov (accessed on 15 July 2021)). Accession numbers: *H. sapiens*: Q9Y2Q0 (*Hs*AT8A1), Q9NTI2 (*Hs*AT8A2), O43520 (*Hs*AT8B1), P98198 (*Hs*AT8B2), O60423 (*Hs*AT8B3), Q8TF62 (*Hs*AT8B4), O75110 (*Hs*ATP9A), O43861 (*Hs*ATP9B), O60312 (*Hs*ATP10A), O94823 (*Hs*ATP10B), Q9P241 (*Hs*ATP10D), P98196 (*Hs*ATP11A), Q9Y2G3 (*Hs*ATP11B), Q8NB49 (*Hs*ATP11C); *Arabidopsis thaliana*: P98204 (*At*ALA1), P98205 (*At*ALA2), Q9XIE6 (*At*ALA3), Q9LNQ4 (*At*ALA4), Q9SGG3 (*At*ALA5), Q9SLK6 (*At*ALA6), Q9LVK9 (*At*ALA7), Q9LK90 (*At*ALA8), Q9SX33 (*At*ALA9), Q9LI83 (*At*ALA10), Q9SAF5 (*At*ALA11), P57792 (*At*ALA12); *Saccharomyces cerevisiae:* P40527 (*Sc*Neo1p), P39524 (*Sc*Drs2p), P32660 (*Sc*Dnf1p), Q12675 (*Sc*Dnf2p), Q12674 (*Sc*Dnf3p); *Caenorhabditis elegans*: Q9U280 (TAT1), U4PBV8 (TAT2), O18182 (TAT3), Q7JPE3 (TAT4), G5EBH1 (TAT5), P91203 (TAT6); *Cryptococcus neoformans*: J9VZ19 (Apt1p), J9VQH2 (Apt2p), J9VGP8 (Apt3p), J9VM87 (Apt4p); *Candida albicans*: A0A1D8PDD2 (*Ca*Neo1p), Q5ADR3 (*Ca*Drs2p), A0A1D8PMY6 (*Ca*Dnf1p), A0A1D8PJN3 (*Ca*Dnf2p), A0A1D8PLQ7 (*Ca*Dnf3p); *Candida glabrata*: Q6FKG3 (*Cg*Q6FKG3), Q6FT10 (*Cg*Q6FT10), Q6FS05 (*Cg*Q6FS05), Q6FST0 (*Cg*Q6FST0), Q6FLT9 (*Cg*Q6FLT9); *Aspergillus nidulans*: Q5ASQ8 (*An*DnfA), Q5B018 (*An*DnfB), Q5BBR9 (*An*DnfC), Q5AYL6 (*An*DnfD); *Aspergillus fumigatus*: Q4WCQ6 (*Af*DnfA), Q4X1T4 (*Af*DnfB), Q4WPR7 (*Af*DnfC), Q4WD94 (*Af*DnfD). *Tremella mesenterica* (NCBI Accessions): *Tm*APT1 (XP_007005544), *Tm*APT2 (XP_007000802), *Tm*APT3 (XP_007003340), *Tm*APT4 (XP_007001948); *Trichosporon asahii*: *Ta*APT1 (J4UDG9), *Ta*APT2 (J5QTD9), *Ta*APT3 (J6ESJ5), *Ta*APT4 (J5T3E8); *Agaricus bisporus*: *Ab*APT1 (K5W6D1), *Ab*APT2 (K5WR21), *Ab*APT3 (K5XCB2), *Ab*APT4 (K5VP00), *Ab*APT5 (K5XG60); *Ustilago maydis*: *Um*APT1 (A0A0D1DYK0), *Um*APT2 (A0A0D1CPW3), *Um*APT3 (A0A0D1DXQ8), *Um*APT4 (A0A0D1C917); *Phanerochaete carnosa*: *Pc*APT1 (K5UY41), *Pc*APT2 (K5VND0), *Pc*APT3 (K5VPX5), *Pc*APT4 (K5VLX9); *Laccaria amethystine*: *La*APT1 (A0A0C9Y7D0), *La*APT2 (A0A0C9WZE4), *La*APT3 (A0A0C9YPB3), *La*APT4 (A0A0C9XZX5), *La*ATP5 (A0A0C9XTN0); *Coprinopsis cinerea*: *Cc*APT1 (A8NE20), *Cc*APT2 (A8NNT2), *Cc*APT3 (A8N6A2), *Cc*APT4 (A8NI63), *Cc*APT5 (A8PGC2); *Dichomitus squalens*: *Ds*APT1 (A0A4Q9N5S3), *Ds*APT2 (A0A4Q9M8M9), *Ds*APT3 (A0A4Q9MCX7), *Ds*APT4 (A0A4Q9M6B7). Sequences were aligned using MUSCLE [46] in MEGA X [47]. The evolutionary history was inferred by using the Maximum Likelihood method and the JTT matrix-based model [48]. Initial tree(s) for the heuristic search were obtained automatically by applying Neighbor-Join and BioNJ algorithms to a matrix of pairwise distances estimated using a JTT model and then selecting the topology with superior log likelihood value.

## 3. Results

### 3.1. C. neoformans Genome Encodes Four Potential P4-ATPases

**Figure 1:**
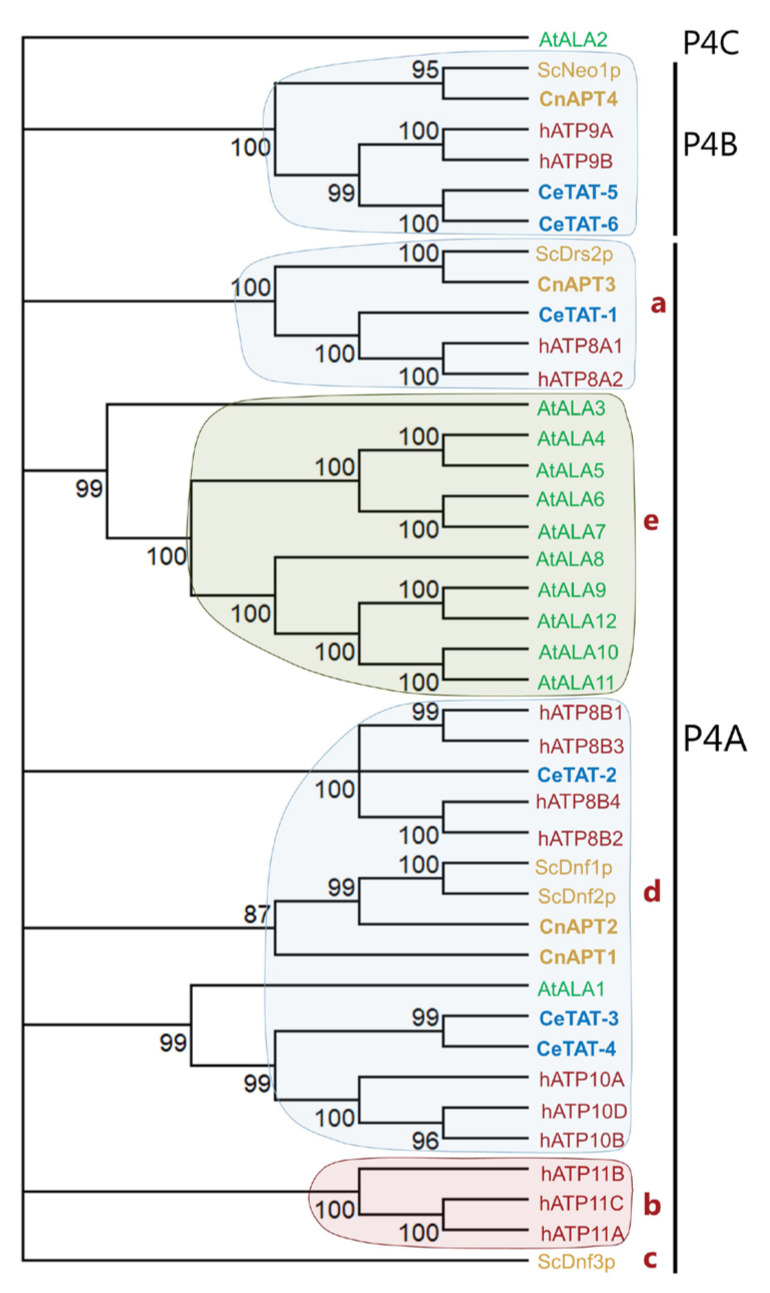
Apt1p belongs to the P4A-ATPase clade. Phylogenetic tree of sequences representing all annotated P4-ATPases from *C. neoformans*, *H. sapiens*, the model plant *A. thaliana*, the yeast *S. cerevisiae*, and the worm *C. elegans*. The phylogenetic tree was inferred from maximum likelihood analysis with 1000 bootstrap iterations performed using Mega X. Node values represent maximum likelihood statistical values with a maximum of 100%. All nodes with statistical values less than 80% were collapsed into multifurcations. In the phylogenetic tree, proteins from humans are written in red, those from plants in green, those from worm in blue, and those from yeast in gold letters. Proteins belonging to the same clade are shadowed: red, human-specific clade; green: plant-specific clade; gold: fungi-specific clade; light blue, clades containing proteins from organisms belonging to several life kingdoms. Shadowing and subclade division within the P4A clade (letters a–e) are as described in [49]. For accession numbers, see Materials and Methods.

Previous sequence analysis of the *C. neoformans* genome identified one *ScCDC50* homolog and four P4-ATPase genes, namely, *APT1*, *APT2*, *APT3*, and *APT4* [37]. Phylogenetic analysis revealed that Apt4p (CnJ9VM87) is a close homolog to *Saccharomyces cerevisiae Sc*Neo1p and can be classified within the P4B-ATPase subfamily, comprising proteins that are functional in the absence of a Cdc50 protein (Figure 1) [49]. All other *C. neoformans* Apt proteins belong to the P4A subclade, characterized by the common requirement for a β-subunit but including P4-ATPases with diverse substrate specificities. Apt1p (CnJ9VZ19) and Apt2p (CnJ9VQH2) are closely related to Dnf1/2p from *S. cerevisiae*, which are broad-specificity transporters capable of recognizing both glycophospholipids and sugar-modified sphingolipids, while Apt3p (CnJ9VGP8) shows homology to PS-transporting *Sc*Drs2p [50]. No homologue of *S. cerevisiae* Dnf3p, which belongs to a subgroup of P4A-ATPases present exclusively in fungi (Figure 1) [49], is found in *C. neoformans* (Suppl. Figure S1).

**Figure 2:**
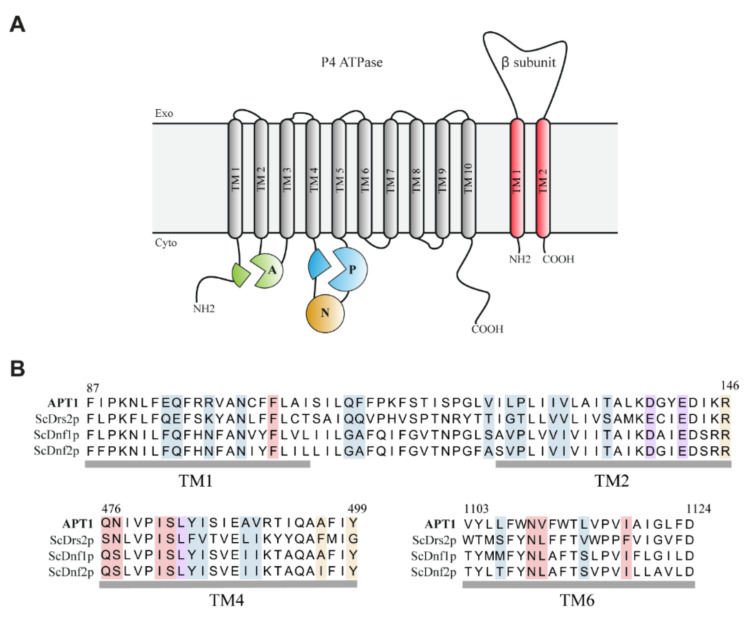
Apt1p belongs to the P4A-ATPase clade. Apt1p harbors functional motifs needed for lipid flipping. (**A**) Membrane topology of P4-ATPases and their β-subunit. P-type ATPases consist of an actuator (A), a phosphorylation domain (P), a nucleotide-binding domain (N), and 10 transmembrane (TM) spanning helices. The Cdc50 subunit consists of two membrane-spanning domains with a large extracellular loop containing four possible N-linked glycosylation sites and two disulfide bridges. (**B**) Alignment of TM1, 2, 4, and 6 of Apt1p and its nearest orthologs in *S. cerevisiae*. The sequences were aligned with Mega X using MUSCLE. Numbers above the alignment represent residues in Apt1p. Gray bars below it represent the transmembrane domains. Residues identified by structural information to be positioned along the lipid translocation pathway are highlighted in blue. Residues shown to be involved in a direct interaction with the lipid are shaded in red for lipid-binding site 1 (loading) and in gold for lipid-binding site 2 (unloading). Residues shaded in purple have been suggested to be required for membrane deformation facilitating lipid loading. For accession numbers, see Materials and Methods. For full sequence comparison, see Supplementary Figure S2.

Our further analysis revealed that Apt1p harbors all functional motifs needed for lipid flipping: a membrane (M) domain with 10 TM helices and a cytosolic region comprising the phosphorylation (P), nucleotide-binding (N), and actuator (A) domains (Figure 2A). Common motifs in the P domain present in Apt1p and found in all P4-ATPases [51] include the DKTGT motif, containing an aspartate that undergoes the phosphorylation–dephosphorylation cycle, as well as the TGDx and GDGxND motifs that bind Mg^2+^ and link the ATP-binding region to the transmembrane (TM) segments. Likewise, the DGET-like motif in the A domain enabling dephosphorylation of the aspartate residue in the DKTGT motif, thereby facilitating lipid flipping, and the PISL-like motif located in the TM4 helix (M domain) and harboring a proline residue involved in protein folding and lipid flipping events [52,53] are present. Determinants of substrate specificity in TM1, 2, 4, and 6 [53,54,55] are partially conserved among the nearest *S. cerevisiae* orthologs *Sc*Dnf1/2p and *Sc*Drs2p (Figure 2B). Out of two glutamine (QQ) residues located in the TM1 entry gate position that is needed for the selection of PS in *Hs*ATP8A1 [16] and *Sc*Drs2 [54], only the first one (Q111) is preserved in Apt1p (Figure 2B, Suppl. Figure S2). Apt1p also harbors residues characteristic for PC/PE/ceramide transporters [16], such as a non-polar residue at position 112 (F112) and a polar uncharged residue at position 476 (Q476). Taken together, we can envisage Apt1p as being a functional lipid flippase.

### 3.2. Heterologous Expression and Localization of Apt1 and Cdc50 Proteins in S. cerevisiae

**Figure 3:**
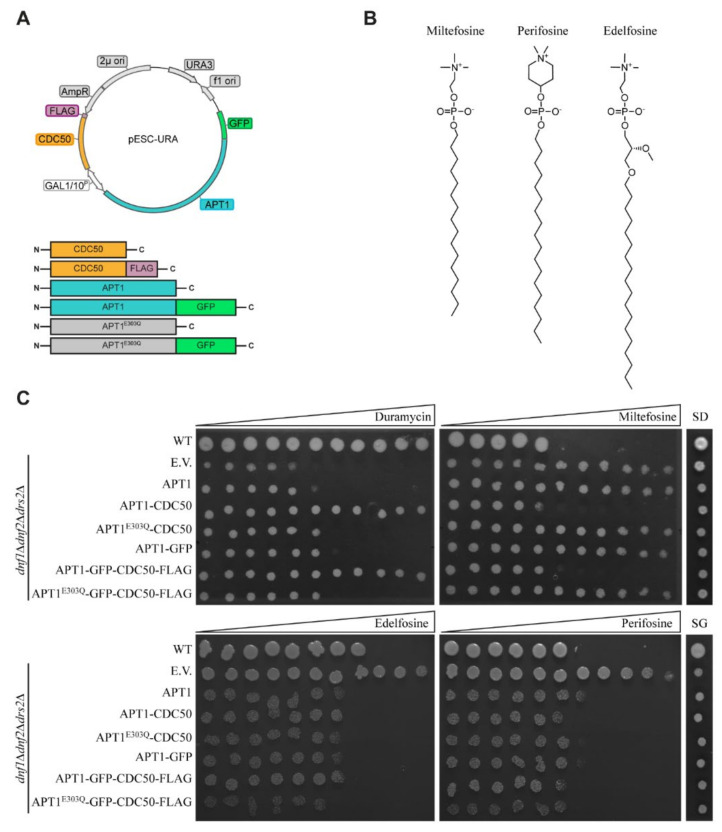
Apt1p/Cdc50p complements the phenotypes of the P4-ATPase-deficient *S. cerevisiae* strain ZHY709. (**A**) A schematic representation of the episomal pESC vectors used to express *APT1* with and without *CDC50* under the control of galactose-induced promoters. (**B**) Chemical structures of edelfosine, miltefosine, and perifosine. (**C**) Functional complementation of a P4-ATPase-deficient yeast strain (ZHY709) bearing wild-type Apt1p and mutant Apt1p^E303Q^ in combination with Cdc50p. Yeast strain BY4741 (wild type) and ZHY709 transformed with empty plasmids (E.V.) were used as a positive and a negative control, respectively. Yeast strains were grown at 30 °C on plates containing a concentration gradient of the indicated toxins (the direction of the gradient is indicated by a triangle) or on control plates with glucose (SD, control) or galactose (SG, induced gene expression) with no further additions. The experiment was repeated at least three times with identical results.

To study the biochemical function of Apt1p, we generated a series of expression plasmids encoding untagged and tagged versions of Apt1p alone or in combination with its potential subunit Cdc50p under the control of a galactose-inducible bidirectional promoter (Figure 3A). In addition, a plasmid encoding a non-functional *APT1* mutant was generated. The corresponding mutant protein lacks the glutamate in a conserved DGET motif (residues 301–304), which is the key amino acid required for dephosphorylation of the P4-ATPase during the catalytic cycle and is consequently blocked in the turnover of the phosphorylated protein. To study the functionality of untagged and tagged proteins, the resulting constructs or the empty vector was used to transform a *S. cerevisiae* mutant (ZHY709). This mutant lacks the endogenous plasma membrane P4-ATPases *Sc*Dnf1p and *Sc*Dnf2p, as well as the Golgi-localized P4-ATPase *Sc*Drs2p, resulting in defective lipid transport and an aberrant exposure of endogenous aminophospholipids at the plasma membrane [14,21,56,57]. Consequently, this mutant is hypersensitive to the membrane impermeable cytotoxic peptide duramycin that binds the aminophospholipid PE. In the presence of this compound, galactose-induced co-expression of *APT1* and *CDC50* (either untagged or tagged) complemented the ZHY709 mutant, whereas cells expressing Apt1p alone or catalytically inactive Apt1p^E303Q^ were not viable (Figure 3C). Miltefosine, edelfosine, and perifosine are alkyl phosphocholine analogues that are toxic only after uptake across the plasma membrane (Figure 3B). Wild-type yeast is sensitive to all three alkyl phosphocholine analogues, whereas ZHY709 is not, due to the lack of the phosphocholine-transporting P4-ATPases *Sc*Dnf1/2p at the plasma membrane (Figure 3B). Upon galactose-induced co-expression of *APT1* and *CDC50*, mutant cells became sensitized to miltefosine, whereas cells bearing Apt1p alone or catalytically inactive Apt1p^E303Q^ remained tolerant (Figure 3C). By contrast, edelfosine and perifosine impaired the growth of mutant cells upon expression of *APT1* variants, and co-expression of *CDC50* did not exert a notable effect. Taken together, these results demonstrate that co-expression of both tagged and untagged versions of Apt1p and Cdc50p partially complemented the phenotypes of the P4-ATPase-deficient *S. cerevisiae* strain ZHY709.

**Figure 4:**
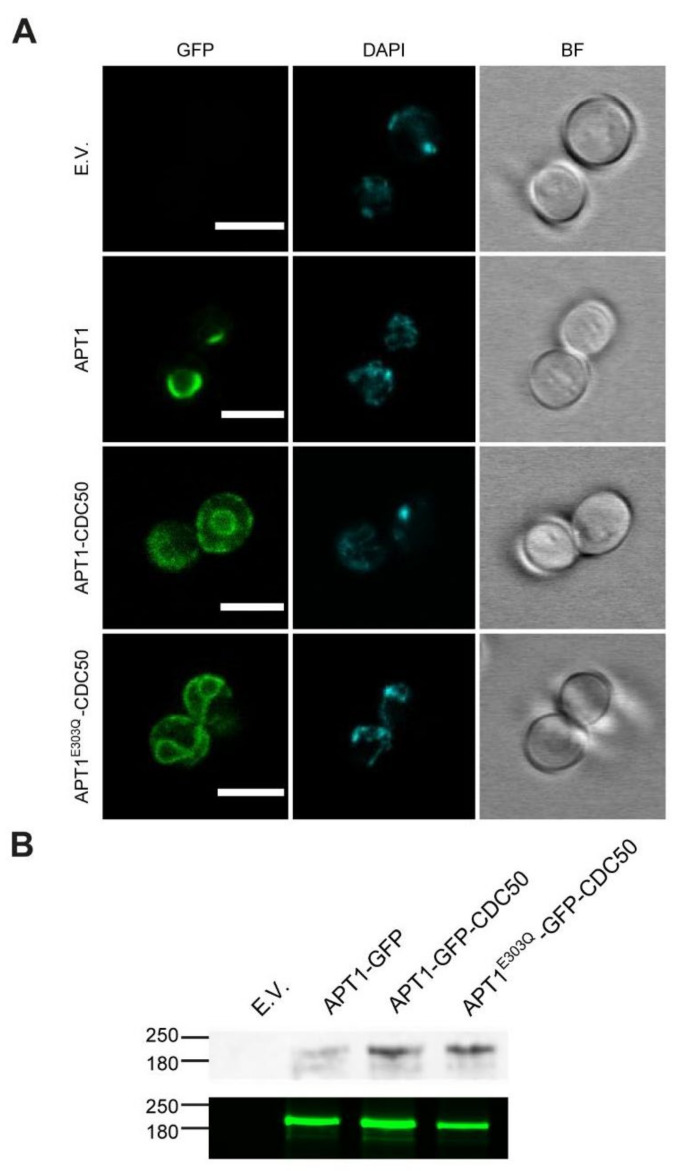
Expression and localization of Apt1p in *S. cerevisiae*. ZHY709 cells were transformed with an empty plasmid vector (E.V.) or with plasmids encoding the indicated Apt1p/Cdc50p variants. Gene expression was induced by growth in galactose medium at 25 °C. (**A**) Representative fluorescence (GFP) or bright-field (BF) confocal images. DAPI (blue) was used to identify nuclei. Scale bars, 5 µm. (**B**) In-gel fluorescence (lower panel) and Western blot analysis (upper panel) of total protein extracts or total membrane preparations. Blots were stained with anti-FLAG antibody. The mobilities of marker proteins of known mass (kDa) are indicated on the left.

Taking advantage of the engineered GFP tags, we studied the subcellular localization of Apt1p and catalytically inactive Apt1p^E303Q^ by confocal fluorescence microscopy (Figure 4). Apt1p-GFP alone displayed a staining pattern characteristic of the yeast ER, with intense labeling of the perinuclear ER (Figure 4A). Upon co-expression with Cdc50p, however, Apt1p-GFP moved from the ER into a more peripheral localization. Apt1p^E303Q^-GFP co-expressed with Cdc50 displayed a localization similar to that observed for wild-type Apt1p-GFP with Cdc50p. Western blot analysis confirmed the expression of both full-length proteins (Figure 4B).

### 3.3. Apt1 and Cdc50 Proteins Form a Stable Complex Showing ATPase Activity

**Figure 5:**
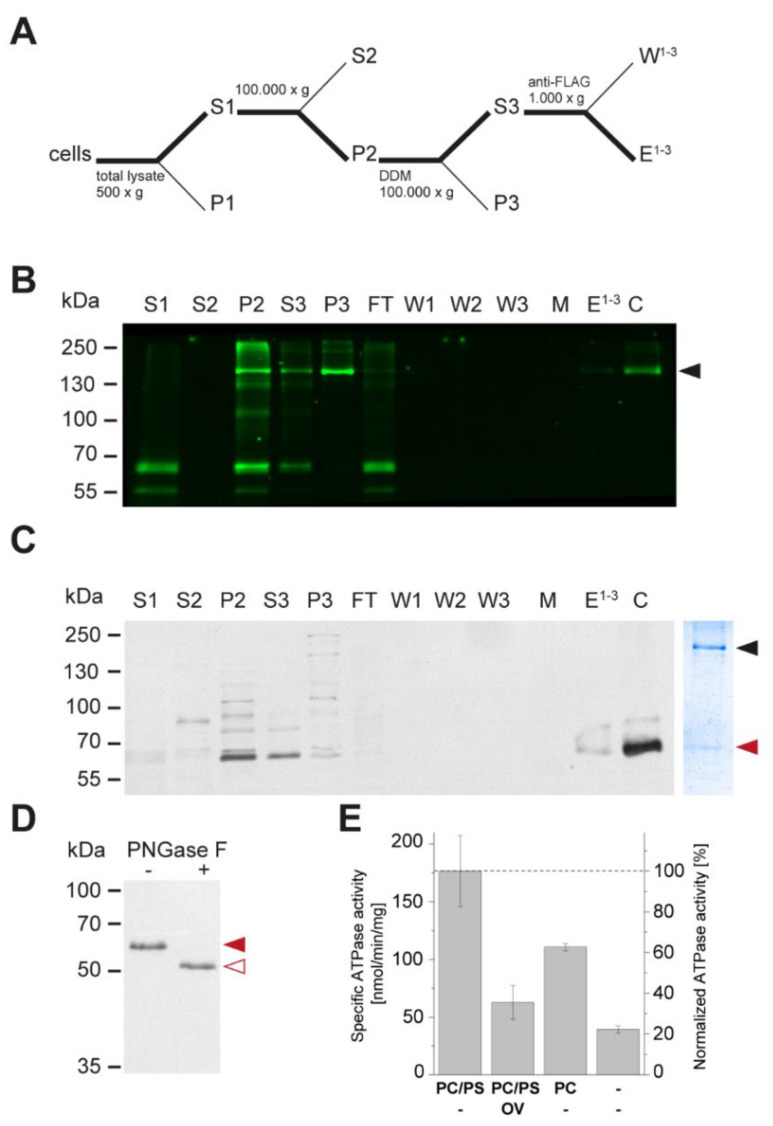
Apt1p and Cdc50p form a stable complex. (**A**) Flowchart of the FLAG-tag affinity purification. Supernatant S1 was centrifuged at 100,000 × *g* (45 min) for collection of total yeast membranes, followed by detergent solubilization and FLAG affinity purification. (**B**) In-gel fluorescence and (**C**) Western blot analysis with anti-FLAG antibodies (**left**) and Coomassie stained SDS–PAGE (**right**) of selected purification fractions. The loaded purification fractions were normalized with respect to volume. (**D**) Deglycosylation assays of Cdc50p. Purified Apt1p-Cdc50p was enzymatically deglycosylated with PNGase F and analyzed by immunoblotting with anti-FLAG antibodies. In panels B, C, and D, filled black, filled red, and non-filled red arrowheads indicate the positions of purified Apt1p-GFP and glycosylated and deglycosylated Cdc50p-FLAG, respectively. The mobilities of marker proteins of known mass (kDa) are indicated on the left. (**E**) ATPase activity of purified Apt1p–Cdc50p assayed in the absence and presence of lipids (PC/PS, molar ratio, 90:10; PC alone) and 1 mM orthovanadate (OV). Results are the means ± S.D. from duplicate determinations (protein with PC/PS/OV, *n* = 3; protein with PC, *n* = 2; protein without lipid, *n* = 1). Abbreviations: S1-3, supernatants; P1-3, pellets; FT, flow-through; W1-3, washes; M, Flag Matrix; E1-3, pooled eluates; C, concentrated eluate. PC, phosphatidylcholine; PS, phosphatidylserine.

To investigate the ability of Apt1p and Cdc50p to form a stable complex, detergent-solubilized membrane preparations obtained from ZHY709 cells expressing *APT1*-*GFP* and *CDC50-FLAG* were subjected to anti-FLAG affinity chromatography, and bound proteins were subsequently eluted by treatment with a buffer containing the FLAG peptide (Figure 5A). This procedure efficiently co-purified Apt1p and Cdc50p (Figure 5B,C; Suppl. Figure S3). Analysis of the Apt1p–Cdc50p complex by SDS–PAGE followed by Coomassie blue staining revealed a band of the expected molecular mass for Apt1p-GFP (~203 kDa), a band at the expected molecular mass for glycosylated Cdc50p-FLAG (~60 kDa), and minor contaminating proteins. Deglycosylation experiments using PNGase F resulted in a single small-molecular-size band (~50 kDa), confirming the glycosylation of Cdc50p-FLAG (Figure 5D). The purified, detergent-solubilized complex displayed maximal ATP hydrolysis in a PC/PS mixture (molar ratio, 90:10), with an apparent ATPase activity of 177 ± 31 nmol ATP min^−1^ mg protein^−1^ (n = 3) (Figure 5E). Sodium orthovanadate inhibited 65% of this activity, indicating that Apt1p–Cdc50p was the dominant active ATPase in the eluate. The protein complex was also active in the presence of PC alone (63% relative to PS/PC), while the absence of added lipids resulted in a basal activity (22% relative to PS/PC). We conclude that Apt1p and Cdc50p form a stable membrane complex showing lipid-stimulated ATPase activity.

### 3.4. Apt1p in Complex with Cdc50p Is a Broad-Specificity Lipid Flippase

**Figure 6:**
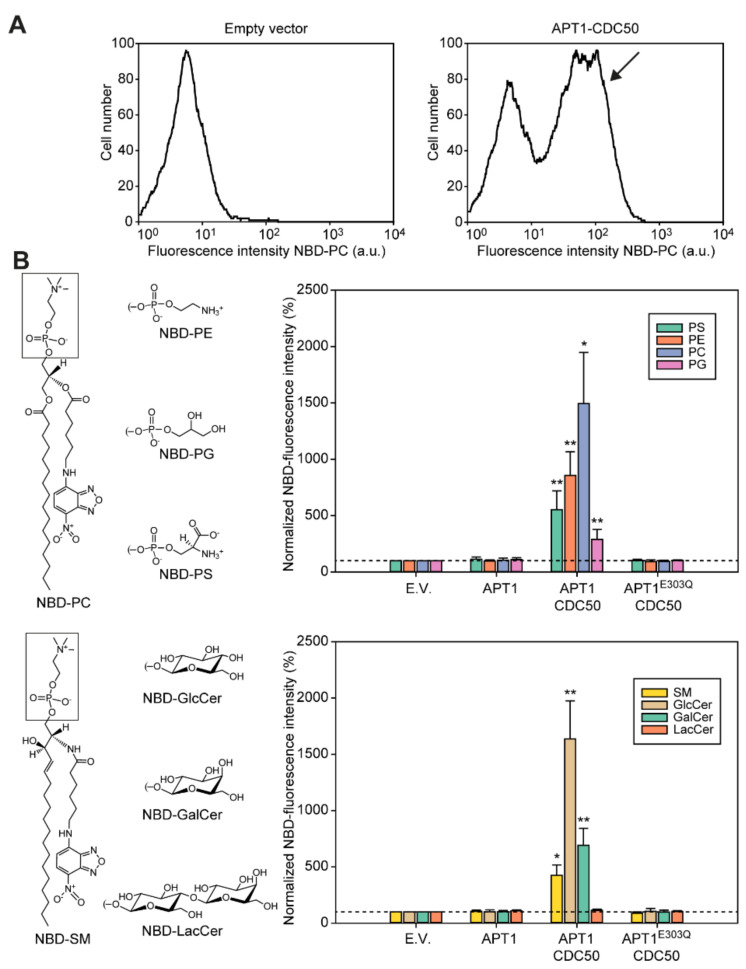
The Apt1p–Cdc50p P4-ATPase complex is a broad-specificity lipid flippase. Internalization of NBD-lipids by P4-ATPase-deficient (ZHY709) cells transformed with an empty vector or plasmids expressing different protein combinations. Yeast cells were labeled with the indicated NBD-lipids, then washed and analyzed by flow cytometry. (**A**) Co-expression of Apt1p and Cdc50p resulted in a population of cells with increased NBD-lipid uptake (arrow). Representative histograms of NBD-PC-labeled cells are shown. (**B**) Structure of the lipids used (**left**; the headgroup region is boxed and attached via a phosphate ester or a glycosidic sugar bond) and bar diagrams (**right**) showing the accumulation of NBD-lipids as a percentage of fluorescence intensity relative to empty-vector (E.V.) controls (set to 100%, dotted line). Values are average ± s.d. of at least three experiments. ** *p* < 0.005 and * *p* < 0.05, significantly different with respect to the E.V. control according to ANOVA analysis with Tuckey confidence test.

We next determined whether co-expression of Apt1p with Cdc50p complemented the lipid internalization defect of the P4-ATPase-deficient *S. cerevisiae* strain ZHY709 by employing fluorescent nitrobenzoxadiazole (NBD) acyl-labelled lipid reporters (Figure 6). To avoid overlapping fluorescent emission signals from NBD and GFP, cells expressing untagged Apt1p were employed. Cells were incubated with different NBD-labelled lipids and, following the removal of the surface-exposed probe, the remaining fluorescence was detected by flow cytometry (Figure 6A). For the quantitative assessment of NBD-lipid internalization, fluorescence intensities were expressed as percentages relative to control cells harboring empty vectors. Co-expression of Apt1p with Cdc50p resulted in a population of cells with increased internalization of a broad range of lipids (Figure 6A). Analysis of the data indicated that the P4-ATPase complex preferentially transported PC, GlcCer > PE > PS, GalCer > SM > PG, but not the diosylceramide lactosylceramide (LacCer) (Figure 6B). Furthermore, a non-functional version of the transporter (Apt1p^E303Q^), in combination with Cdc50p, was unable to support the uptake of fluorescent lipids, confirming that P4-ATPase activity is a requirement for lipid internalization.

## 4. Discussion

Previous studies revealed overlapping functions of both the P4-ATPase Apt1p and the Cdc50 protein in the maintenance of membrane integrity, implying that Cdc50p serves as a β-subunit for Apt1p to form a functional lipid flippase [36,37,39,58]. By using heterologous expression in *S. cerevisiae*, the present study provides direct evidence that both proteins form a stable heterodimeric complex resulting in a functional lipid flippase. These findings are consistent with Cdc50 proteins being an integral part of the P4A-ATPase lipid flippases. In *S. cerevisiae*, the Cdc50 family members *Sc*Cdc50p, *Sc*Lem3p, and *Sc*Crf1p form stable complexes with *Sc*Drs2p, *Sc*Dnf1p/Dnf2p, and *Sc*Dnf3p, respectively [59,60]. Formation of these complexes is required for proper expression and ER export of either partner [59,60,61]. In agreement with this notion, we observed ER retention of Apt1p in the absence of Cdc50p expression. In all eukaryotic genomes examined so far, P4-ATPase genes greatly outnumber *CDC50* genes, suggesting that a given Cdc50 protein can interact with multiple P4-ATPases. This notion is supported by studies on fungal, human, and plant P4-ATPases. In *S. cerevisiae*, *Sc*Lem3p does associate with both P4A-ATPases *Sc*Dn1p and *Sc*Dnf2p. Likewise, the human P4A-ATPases *Hs*ATP8B1, *Hs*ATP8B2, *Hs*ATP8B3, *Hs*ATP8A1, and *Hs*ATP8A2 each interact with either *Hs*Cdc50A or *Hs*Cdc50B [13,62], and various *Arabidopsis* Cdc50p homologs form stable complexes with several plant P4A-ATPases [14,63,64]. Conceivably, *C. neoformans* Cdc50p may also serve as a binding partner for the P4A-ATPases Apt2p and Apt3p. Apt4p, as a P4B-ATPase, might not require a β-subunit to function, as known for the P4B-ATPase *Sc*Neo1p [35,61] and *Hs*ATP9B [65]. The heterologous expression system utilizing *S. cerevisiae* mutants presented here may help clarify this issue.

Complementation tests and uptake assays employing fluorescent lipids served as an approach to analyze lipid transport activity of the Apt1p-Cdc50p complex. We found that the Apt1p–Cdc50p complex facilitates the uptake of a broad lipid spectrum, including several phospholipids and monoglycosylceramides. The physiological relevance of this broad substrate specificity remains to be established but is reminiscent of the broad substrate specificity of the two plasma membrane P4A-ATPases Dnf1/2p in *S. cerevisiae* [29,30]. In the heterologous expression model used here, co-expression of Apt1p and Cdc50p reversed an aberrant exposure of endogenous aminophospholipids, as evidenced by reduced hypersensitivity of the cells to the PE-binding peptide duramycin. These results are in line with a direct role of the Apt1p–Cdc50p complex in pumping natural PE to the cytosolic leaflet to generate an asymmetric membrane, as recently suggested for P4-ATPases from other organisms [5,66]. The ability of the Apt1p–Cdc50p complex to transport NBD-PC and NBD-PS suggests that natural PC and PS are maintained in the cytoplasmic leaflet and that the outer leaflet is primarily composed of higher sphingolipids. In line with this notion, *C. neoformans* mutant cells lacking *CDC50* expose PS on the cell surface [40]. The dynamic regulation of transbilayer lipid asymmetry by P4-ATPases has been found to be crucial for actin organization and membrane transporter function [25]. Thus, several phenotypes observed for *C. neoformans* mutant cells lacking *CDC50* or *APT1* might be caused by a disturbed lipid membrane asymmetry. Further studies are required to identify the subcellular localization of Apt1–4p in *C. neoformans* to dissect their individual cellular roles.

Notably, Apt1p in complex with Cdc50p was found to transport the monoglycosylceramides GlcCer and GalCer. GlcCer is an important regulator of cryptococcal virulence [67]. This neutral glycosphingolipid is found at the fungal cell wall [68] and in extracellular vesicles [69]. An alteration in the membrane lipid structure may result in an altered raft formation, thereby affecting fungal membrane fluidity and rigidity [70]. Thus, Apt1p might be needed for a tight regulation of the GlcCer contents of the cellular membranes and cell wall. In line with this concept, deletion of *APT1* affects intracellular membrane organization and results in altered lipid metabolism, with reduced levels of GlcCer and inositol phosphoryl ceramides in *C. neoformans* [38].

Apt1p might also serve to scavenge GlcCer from the host. In the yeast *S. pombe*, the *Sp*Dnf2p ortholog transports GlcCer and GalCer but not PC and PE, suggesting that glycosphingolipid transport is a consolidated function of *Sp*Dnf2p [30]. *S. pombe* is unable to synthesize GlcCer, and it has been hypothesized that P4-ATPase-mediated transport of GlcCer in this organism serves a role in scavenging this lipid from the plant material the yeast grows upon [30,71]. The interconnections between Apt1p and GlcCer flipping are intriguing, since both molecules are required for both pathogenic and secretory mechanisms. This implies that lipid transport in *C. neoformans* is finely regulated to promote fungal virulence, which reinforces the potential of both Apt1p and GlcCer as targets for antifungal drug development [68,72].

In conclusion, our work identifies Apt1p in complex with Cdc50p as a lipid flippase with broad substrate specificity. This information represents a first step towards the understanding of the essential role of this transporter during infection and towards its validation as a drug target. The availability of a heterologous expression system for Apt1p–Cdc50p may prove useful for the expression and purification of the complex for further detailed biochemical analysis aimed at identifying novel antagonists of this transporter class that might help in the treatment of cryptococcal meningitis and related infections in humans.

## Data Availability

Flow cytometry data are available upon request from the corresponding author. All other data are available within the article and Supplementary Materials.

## Supplementary Materials

The following are available online at https://www.mdpi.com/article/10.3390/jof7100843/s1, Figure S1. Phylogenetic relations amongst fungal P4 ATPases. Figure S2. Sequence alignment of Apt1p and its nearest orthologs in *S. cerevisiae.* Figure S3. Quantification and visualization of the purified Apt1p-GFP/Cdc50a-FLAG. Table S1. Primers used in this study.
