# Supplementary material for: P-Type ATPase Apt1 of the Fungal Pathogen Cryptococcus neoformans Is a Lipid Flippase of Broad Substrate Specificity
Source: J Fungi (Basel). 2021 Oct 8;7(10):843. doi: 10.3390/jof7100843 (PMC8537059; doi:10.3390/jof7100843)
Supplement: Supplementary file 1 [file jof-07-00843-s001.zip › jof-1342704-supplementary.pdf]

P-type ATPase Apt1 of the fungal pathogen *Cryptococcus neoformans* is a lipid flippase of broad substrate specificity

Lyubomir Dimitrov Stanchev [1,2], Juliana Rizzo [3], Rebecca Peschel [1], Lilli Pazurek [1], Lasse Bredegaard [2], Sarina Veit [1], Sabine Laerbusch [1], Marcio L. Rodrigues [3,4], Rosa L. López-Marqués [2], Thomas Günther Pomorski [1,2,*]

Supplementary Information

This file includes all supplementary information for the manuscript:

- Figures S1, S2 and S3

- Tables S1

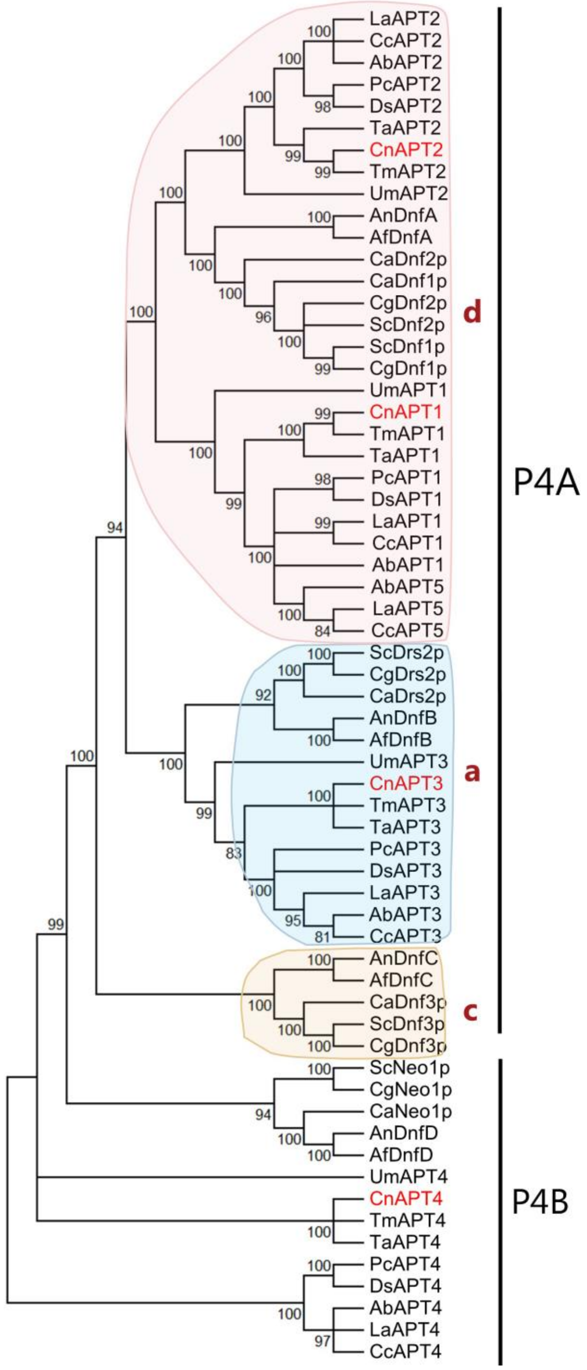

Supplementary Figure S1. Phylogenetic relations amongst fungal P4 ATPases. Phylogenetic tree of sequences representing all annotated P4-ATPases from selected yeast and fungi: *Saccharomyces cerevisiae* (*Sc*), *Cryptococcus neoformans* (*Cn*); *Candida albicans* (*Ca*), *Candida glabrata* (*Cg*), *Aspergillus nidulans* (*An*), *Aspergillus fumigatus* (*Af*), *Tremella mesenterica* (*Tm*), *Trichosporon asahii* (*Ta*), *Agaricus bisporus* (*Ab*), *Ustilago maydis* (*Um*), *Phanerochaete carnosa* (*Pc*), *Laccaria amethystine* (*La*), *Coprinopsis cinerea* (*Cc*), *Dichomitus squalens* (*Ds*). The phylogenetic tree was inferred from maximum likelihood analysis with 1,000 bootstrap iterations performed using Mega X. Node values represent maximum likelihood statistical values with a maximum of 100%. All nodes with statistical values less than 80% were collapsed into multifurcations. In the phylogenetic tree, proteins from *C. neoformans* are indicated in red. Colored balloons indicate proteins that belong to the same phylogenetic cluster (small dark red letters), as described in [Palmgren et al., 2019, Biochim Biophys Acta Biomembr. 1861:1135-1151]. For accession numbers, see Materials and methods.

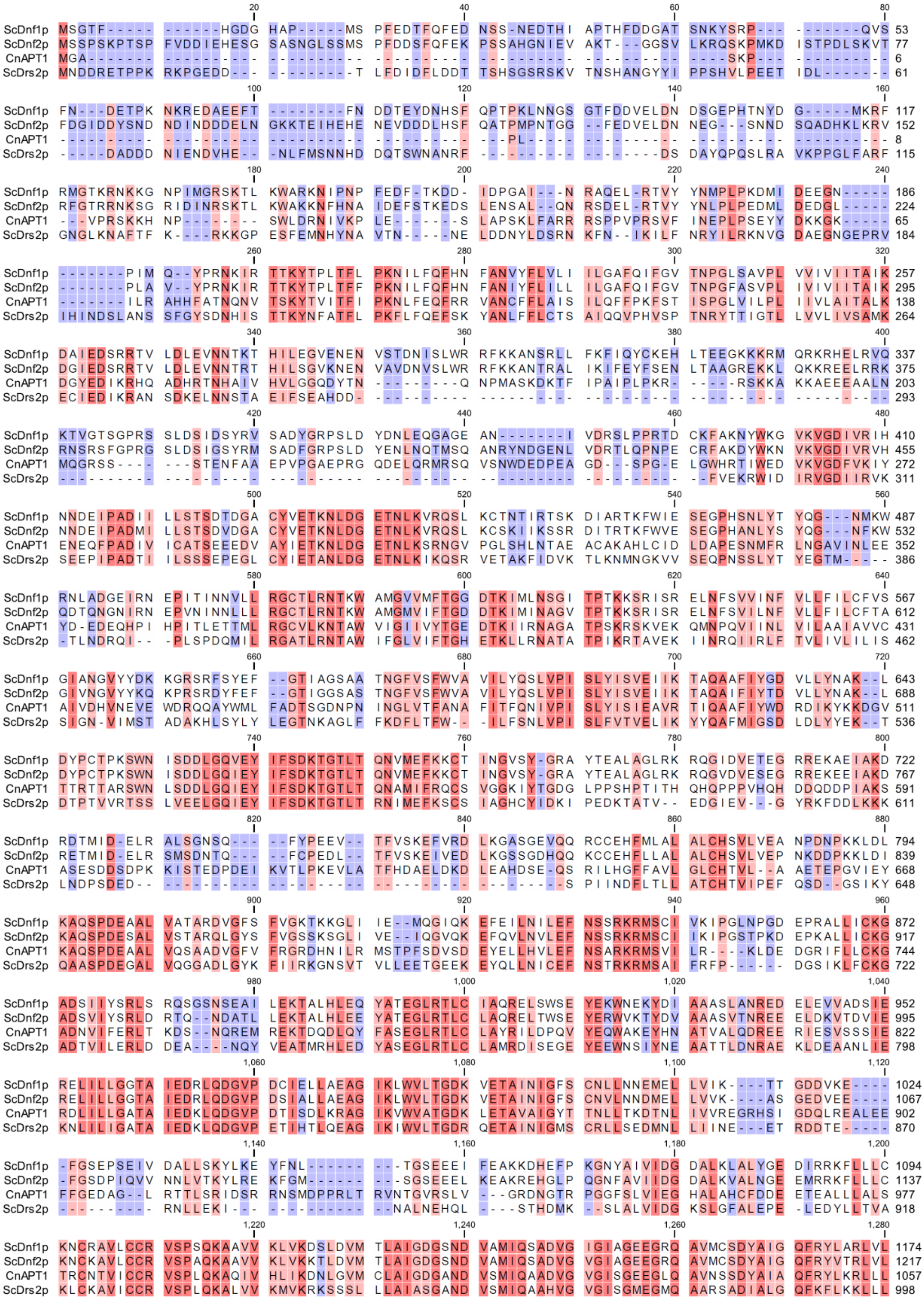

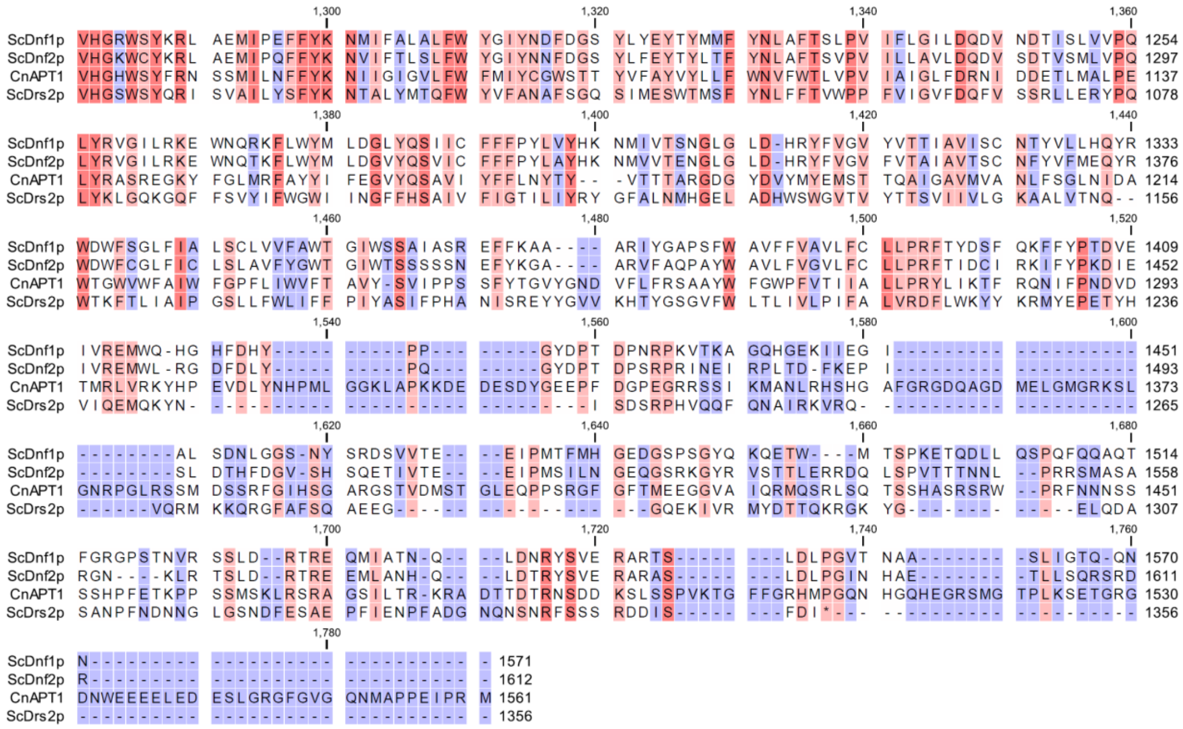

Supplementary Figure S2. Sequence alignment of Apt1p and its nearest orthologs in *S. cerevisiae*. The sequences were aligned with Mega X using MUSCLE. Positions are coloured blue to red according to increasing sequence identity. For accession numbers, see Materials and methods.

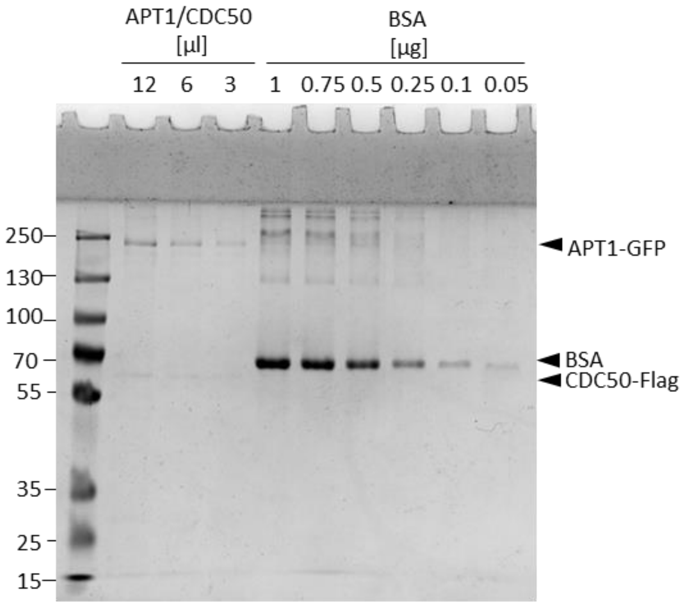

Supplementary Figure S3. Quantification and visualization of the purified Apt1p-GFP/Cdc50a-FLAG. Purified Apt1p-GFP/Cdc50a-FLAG were subjected to SDS-PAGE followed by Coomassie Brilliant Blue staining. By comparing the band intensity of the purified protein with that of BSA, the concentration of Apt1p-GFP was estimated to be approximately 6.4 ng μl^{-1}. Molecular mass markers (M) and the molecular mass (kDa) are shown on the left. Band intensities were quantified using Image LabTM Software from Bio-Rad.

Supplementary Table S1. Primers used in this study.

Set	Name	Sequence
#1	*APT1_F*	5'-AAAAAACCCCGGATCCATGGGCGCCTCCAAACC-3'
	APT1_R	5'-GTCGTATTACGGATCCTTACATCCTCGGTATTTCTGGTGGG -3'
#2	*Spacer-myc-for*	5'-GGTTCTGGTTCTGAACAGAAGTTGATTTCCG-3'
	Spacer-myc-rev	5'-AGAACCAGAACCCATCCTCGGTATTTCTGG-3'
#3	*APT1_Frag. 1. FOR*	5'-AAAAAACCCCGGATCCATGGGCGCCTCC-3'
	APT1-spacerGFP-rev	5'-CTCACCATAGAACCAGAACCAGAACCAGAACCC -3'
#4	*spacer-eEGFP-for*	5'-TGGTTCTATGGTGAGCAAGGGCGAG-3'
	eGFP-rev	5'-GTCGTATTACGGATCCTTACTTGTACAGCTCGTCCATGCC -3'
#5	*CDC50_F*	5'-CACTAAAGGGCGGCCGCATGGCCATATTCAACAGGAAGCC-3'
	CDC50_R	5'-GATACTAGTGCGGCCGCTTATAATCCATTTGCGTTGGGCTGGT-3'
#6	*CDC50_F*	5'-CACTAAAGGGCGGCCGCATGGCCATATTCAACAGGAAGCC-3'
	CDC50_R without Stop	5'-GATACTAGTGCGGCCGCTAATCCATTTGC-3'
#7	*Dead-Mut for*	5'-CCTCGACGGTcAAACCAACTT-3'
	Dead-Mut rev	5'-TTTTTGGTTTCGATGTACGC-3'
